# Assessment of Robotic Patient Simulators for Training in Manual Physical Therapy Examination Techniques

**DOI:** 10.1371/journal.pone.0126392

**Published:** 2015-04-29

**Authors:** Shun Ishikawa, Shogo Okamoto, Kaoru Isogai, Yasuhiro Akiyama, Naomi Yanagihara, Yoji Yamada

**Affiliations:** 1 Department of Mechanical Science and Engineering, Graduate School of Engineering, Nagoya University, Nagoya, Japan; 2 Department of Physical Therapy, Faculty of Health and Medical Science, Tokoha University, Hamamatsu, Japan; 3 Department of Physical and Occupational Therapy, Graduate School of Medicine, Nagoya University, Nagoya, Japan; Peking University, CHINA

## Abstract

Robots that simulate patients suffering from joint resistance caused by biomechanical and neural impairments are used to aid the training of physical therapists in manual examination techniques. However, there are few methods for assessing such robots. This article proposes two types of assessment measures based on typical judgments of clinicians. One of the measures involves the evaluation of how well the simulator presents different severities of a specified disease. Experienced clinicians were requested to rate the simulated symptoms in terms of severity, and the consistency of their ratings was used as a performance measure. The other measure involves the evaluation of how well the simulator presents different types of symptoms. In this case, the clinicians were requested to classify the simulated resistances in terms of symptom type, and the average ratios of their answers were used as performance measures. For both types of assessment measures, a higher index implied higher agreement among the experienced clinicians that subjectively assessed the symptoms based on typical symptom features. We applied these two assessment methods to a patient knee robot and achieved positive appraisals. The assessment measures have potential for use in comparing several patient simulators for training physical therapists, rather than as absolute indices for developing a standard.

## Introduction

Manual examination is one of the essential techniques employed in physical therapy. A physical therapist manually tests the biomechanical and neural responses of the limbs of a patient to understand their pathology and the status of the disorder. In the application of this technique, the therapist examines the dynamic joint-resistances, ranges of joint motions, and complaints of pain by the patient when the impaired limb is passively moved. The therapist requires clinical experience to master the manual examination procedures because the joint resistance is highly dependent on the individual patient and specific disease. However, in educational facilities, trainee therapists hardly get the opportunity to deal with real patients. Even during internship, engaging trainees in the treatment of patients is considered ethically unacceptable.

To solve such problems in educational facilities, some research groups have recently conducted studies on patient robots that can be used to aid the training of physical therapists in manual examination techniques. For example, Masutani et al. [1] developed a leg robot that simulates the symptoms of joint contracture and limited joint motion. Kikuchi et al. [2, 3] arranged two types of actuators on a leg robot to simulate both large and quick force outputs. Morita et al. [4] also developed a leg robot with a two-degree-of-freedom knee joint that rotates when it flexes or extends in imitation of the behavior of a human knee. Grow et al. [5] and Tkahashi et al. [6] developed elbow robots for similar educational purposes. Mouri et al. [7] imitated finger contracture using a robotic hand with 22 actuated joints. Human peculiarities were taken into consideration in the development of some of these robots, including complex human joint motions, which are intrinsically different from those of mechanical hinges and universal joints, and the skin and bone features of human limbs. This was because such realism is instrumental to the proper learning of manual examination techniques by trainees. However, the developers also considered striking an appropriate balance between the realism and economy of the patient robots. In the quest for a well-balanced patient simulator, Ishikawa et al. [8, 9] and Okumura et al. [10] introduced the concept of wearable patient robots. In this case, healthy students wear exoskeleton robots on their legs or feet, and actuators installed on the robots produce resistance forces to mimic impaired joints. The trainees can thus have firsthand experience of the disorders while learning the manual examination techniques.

However, these previous works did not systematically validate the simulated symptoms, although there were assertions of corroboration by clinicians. For example, in [3], there was the question of whether the specific parameters used to characterize the simulated symptoms, such as the maximum joint torque, were within the range of those of actual patients. As a sanity check, some works either commented on the apparent behaviors of the simulated outputs [4] or rated the subjective realism [7]. In addition, these earlier studies did not employ the perception of clinicians in the statistical examination of how well the symptoms were simulated. However, Park et al. [11] and Kim et al. [12], in their thorough examination of the validity of their developed robotic elbow spasticity simulators, requested clinicians to rate both a real patient and the simulated elbow spasticity using a well-known clinical spasticity criterion. They then discussed how well the clinicians’ assessments of the real and simulated disorders matched.

Because effective evaluation methods are yet to be established at this early stage of the development of patient simulators used for physical therapy training, they have not been thoroughly validated. One possible evaluation method involves comparison of simulated joint resistances with those measured from real patients [11]. However, the data obtained from patients contain significant individuality, which makes the approach more suitable for the further training of experienced clinicians. Considering the nature of a typical lecture scene at an educational facility, a patient robot is required to reproduce or simulate typical symptoms rather than those unique to individual patients. Hence, Fujisawa et al. [13] considered the typical resistance of a spastic elbow in their study, wherein an experienced physical therapist mimicked the typical behaviors of a disabled elbow by using his own elbow, and the robotic patient simulator was then designed to reproduce those behaviors. Based on the notion that patient robots used for educational purposes simulate generalized symptoms that majority of clinicians consent to, we designed methods for evaluating patient robots used for physical therapy training. We statistically assessed the extent to which a robot demonstrated symptoms that were considered invariant and typical by clinicians, whereas most previous studies were concerned with the error of the torque outputs in the simulation of specific actual patients. Moreover these previous assessments were not based on the perceptions of practicing clinicians, and most of them also employed non-statistical analyses. To the best of our knowledge, only few studies have statistically assessed such patient robots from the perspective of generalized symptoms.

Our is thus the first article that would propose general measures for assessing patient robots used to train physical therapists in manual examination techniques. Two types of assessments that we propose are based on the judgment of clinicians, and were experimentally applied to our patient robot. The two assessment types complement each other by addressing distinct aspects of patient simulators, and their measures can be used as practical indices for comparing different patient simulators when they become commercially available in the near future. There are currently no such performance indices. All the experimental procedures of the present study were approved by the Ethics Committee, School of Engineering, Nagoya University, and each participant signed an informed consent document.

**Severity assessment of specific disease** The first measure is the evaluation of how well the patient robot presents the severity of a specific disease, and is based on [9]. Experienced therapists were requested to judge the severity of the simulated symptoms using a clinical rating method, and we then examined the goodness of the simulated symptoms based on the variance or consistency of the judgments of the therapists. If the symptoms were well simulated, there would be fair agreement among the judgments. This approach is basically similar to that adopted by Park et al. [11], although they used an elbow robot that mimicked the joint resistances of three real patients, and not generalized symptoms.

**Disease classification ability** The second measure is the evaluation of how well the patient robot presents the symptoms of different diseases. The therapists were requested to classify several simulated symptoms, and we examined the rates of successful classifications. The higher the average classification rates, the more accurately the simulator presented the typical features of different symptoms.

## Wearable robotic knee joint as impairment simulator

**Fig 1:**
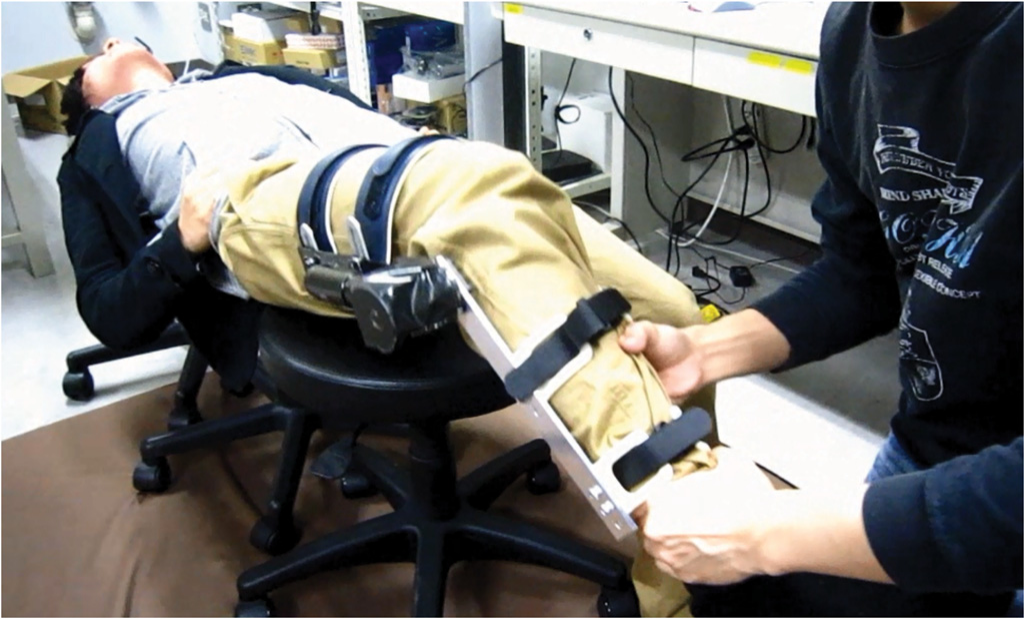
Training in manual examination techniques using a wearable knee joint. Left: Healthy person wearing the robotic knee joint. Right: Trainee therapist

As shown in Fig 1, we used a wearable robot [8, 9] to simulate the resistance forces of the knee of a patient. In physical therapy educational facilities, trainees commonly study manual examination techniques in pairs. One trainee acts as a patient and the other practices the techniques. The wearable patient robot is particularly designed for such training situations. The trainee that plays the part of a patient wears the robot and relaxes to avoid generating voluntary forces. The other trainee manually tests the dummy patient leg for which the robot produces a resistance force to simulate joint impairments such as abnormal muscular tensions or neural disorders.

**Fig 2:**
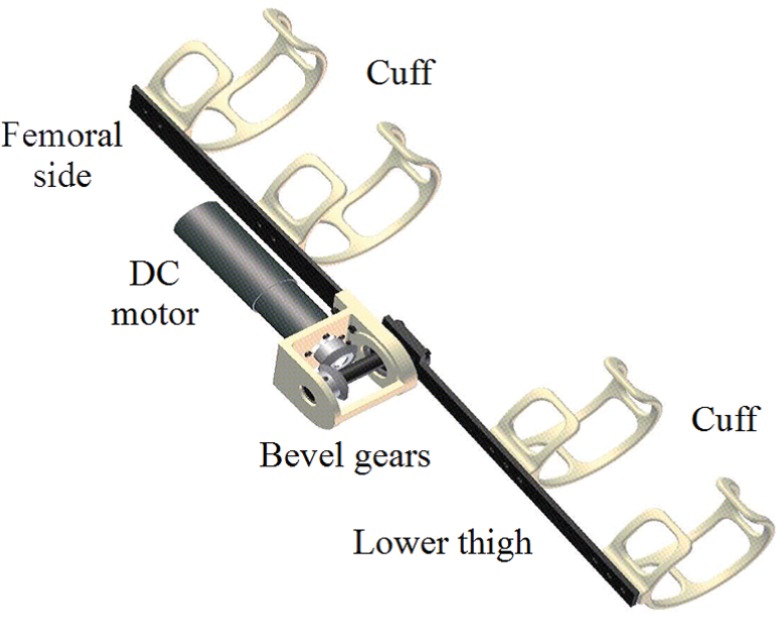
Structure of wearable knee joint.

As shown in Fig 2, the wearable robot has a geared (1/86) DC motor (RE35, Maxon, stalling torque = 949 mN⋅m), encoder, two links, and four cuffs for attachment to the femoral and lower thighs of the wearer. The DC motor was activated by a current-control motor driver (4-Q-DC ADS 50/5, Maxon). The gear friction was fairly constant and compensated for during the motion. The total weight of the device was 1.16 kg. Although natural human knee motion involves minor stretch and rotation, which contrasts with that of the uniaxial robot, the compliance of the skin or clothes beneath the cuffs obscures the mechanical differences between the two.

The advantages of a wearable patient simulator include the enablement of trainees to touch and feel an actual human limb, thereby affording the perception of the complex motion of the human joint and the features of the human skin and bone. This concept provides high realism at relatively low costs. However, it also has some limitations, which include the fact that the symptoms that can be simulated by the wearable simulator are physically limited by the body characteristics of the wearer. For example, a knee joint significantly deformed by osteoarthritis cannot be simulated by a healthy wearer. Furthermore, the mass and inertia moment of the wearable device cannot, in principle, be completely compensated for. In the case of the knee joint, if the hip joint of the wearer is fixed, his femoral thigh would receive the torques used to compensate for the mass and inertia of the robot. However, this is not always the case during a manual examination. In the present study, the mass and inertia moment were not canceled.

## Severity assessment of simulated spasticity

In this assessment type, we evaluated how well the patient robot presented differing severities of a particular type of disease. We developed a resistance model of a spastic joint and simulated several degrees of severity using the patient simulator. Practicing physical therapists were requested to rate the severities based on the common symptoms of spasticity. We then examined the consistency of the ratings of the therapists and how well the spasticity was simulated.

### Spasticity and modified Ashworth scale

Spasticity is typically triggered by stroke or injury to the spinal cord, brain, or other central nervous system lesions [14]. It is a motor disorder with a velocity-dependent joint resistance that increases in the tonic stretch reflex, with exaggerated tendon jerks resulting from hyperexcitability of the stretch reflex [15, 16]. Approximately 40% of stroke survivors develop symptomatic spasticity in their chronic phases [17, 18].

The modified Ashworth scale (MAS) [19] is a well-known clinical criterion of spasticity. As presented in Table 1, it can be used to classify the severity of spasticity into six levels based on the resistances that a physical therapist experiences when moving the joints of the patient. For example, at level 1, mild muscle tone is palpable with a sense of slight sticking during passive joint movement. Additionally, weak resistance manifests at the end of the range of motion. When the severity increases to level 2 or 3, increased muscle tones are observable through most of the range of motion. Finally, at level 4, the joint is completely rigid and cannot be moved.

**Table 1:** Modified Ashworth scale as described in the source article [19].

Score	Modified Ashworth Scale
0	No increase in muscle tone
1	Slight increase in muscle tone, manifested by a catch and release or by minimal resistance at the end of the range of motion when the affected part(s) is moved in flexion or extension
1+	Slight increase in muscle tone, manifested by a catch, followed by minimal resistance throughout the remainder (less than half) of the range of movement (ROM)
2	More marked increase in muscle tone through most of the ROM, but affected part(s) easily moved
3	Considerable increase in muscle tone, passive movement difficult
4	Affected part(s) rigid in flexion or extension

### Simulated spasticity

Velocity-dependent tonic resistance is often considered as a characteristic feature of spastic joints. However, as indicated in Table 1, muscle and tendon rigidity [20] and limitation of the range of motion are also practical criteria for evaluating the extent of the symptoms. Our dummy joint collectively simulated these criteria symptoms.

The phasic stretch reflex torque [15, 16] is commonly expressed in terms of the viscosity resistances with respect to the joint angular velocity [2, 5, 11, 20]. The resistance torque is considered to be negligible up to a particular angular velocity, *ω*_0_, which corresponds to the dynamic stretch reflex threshold, namely the velocity-dependent joint angle at which stretch reflex manifests (e.g., [21]). The threshold was originally defined as a line on a phase-plane of the joint angle and angular velocity. The reflective resistance thereafter increases proportionally to the angular velocity. If the joint angle is denoted by *θ*(*t*) and the coefficient of viscosity by *c*, the velocity-dependent resistance torque *τ*_*c*_(*t*) can be expressed as

$$\begin{array}{c} \tau_{c}\left(t\right)=\{\begin{array}{cc} c(\dot{\theta }\left(t\right)-\omega_{0}) & \left(\dot{\theta }\left(t\right)>\omega_{0}\right)\\ 0 & (\dot{\theta }\left(t\right)\leq \omega_{0}). \end{array} \end{array}$$ (1)

The resistance acts in only one direction because, typically, either the flexors or the extensors are affected by such diseases. The equation gives the resistance in only the flexion phase when *θ* = 0 at full extension.

According to the MAS, a limit in the range of motion manifests from level 1. At level 1+, there is resistance to over nearly half of the range of motion. When the range of motion of the knee joint is 0 < *θ*(*t*) < *θ*_max_, the resistance torque at the end of the motion, *τ*_*e*_(*t*), is given by

$$\begin{array}{c} \tau_{e}\left(t\right)=\{\begin{array}{cc} 0 & \left(0\leq \theta \left(t\right)<\theta_{\max}-\theta_{e}\right)\\ k_{e}\left(\theta \left(t\right)-\theta_{\max}+\theta_{e}\right) & (\theta_{\max}-\theta_{e}\leq \theta \left(t\right)\leq \theta_{\max}), \end{array} \end{array}$$ (2)

where *k*_*e*_ and *θ*_e_ denote the modulus of elasticity and the angle at which limitation of the joint motion begins, respectively. These parameters increase with the severity of the spasticity. We also introduced the constant resistance *τ*_r_, which acts throughout the entire motion range. Consequently, the total resistance is

$$\begin{array}{c} \tau \left(t\right)=\tau_{r}+\tau_{c}\left(t\right)+\tau_{e}\left(t\right). \end{array}$$ (3)

The five parameters *τ*_r_, *ω*_0_, *c*, *θ*_e_, and *k*_e_ were varied to reflect the severity of the spasticity.

### Severity rating of simulated spasticity

#### Tasks and participants

A healthy person wore the wearable robot on his right leg and lay down on a clinical bed. The assessor freely tested the supposed knee impairment by moving the leg of the wearer and rated the severity of the simulated spasticity based on the six MAS levels. Each trial was limited to within 1 min. After all the tasks, we asked the assessor about the subjective criteria that they particularly considered in rating the simulated symptoms.

Eight practicing physical therapists, each with more than three years of clinical experience, participated in the experiment after a few minutes of practice without the simulated symptoms. None of them had previously experienced simulated spasticity.

#### Parameter sets of simulated spasticity

We varied five parameters in presenting different spastic resistances as enumerated in Table 2. Ten spasticity grades were prepared, namely, *S*_0_–*S*_9_, with *S*_0_ being the least severe (healthy state). For *S*_9_, *τ*_r_, *ω*_0_, *c*, *θ*_e_, and *k*_e_ were set to 3.0 N ⋅ m, 0 rad/s, 9/*π* Nm ⋅ s/rad, 16*π*/45 rad, and 54/5*π* N ⋅ m/rad, respectively. These parameters were specified with the assistance of two physical therapists, K. I. and N. Y., who are coauthors of this article, such that the majority of physical therapists would likely regard *S*_9_ as 3 on the MAS. We excluded the severest MAS level at which the joint is almost unmovable because the DC motor on the wearable robot could not generate sufficient torques to fully resist the manual forces applied by the assessors. The parameters for *S*_0_ were set such that no resistance was generated. Regarding the other eight simulated spasticity conditions, each of the parameters was linearly varied between the maximum and minimum values in Table 2. The simulated spasticity conditions were presented in random order, and two different orders were used for each participant.

**Table 2:** Parameter sets of simulated spasticity.

	Simulated spasticity									
	*S*_0_	*S*_1_	*S*_2_	*S*_3_	*S*_4_	*S*_5_	*S*_6_	*S*_7_	*S*_8_	*S*_9_
*τ*_r_ [N⋅m]	0	0.3	0.7	1.0	1.3	1.7	2.0	2.3	2.7	3.0
*ω*_0_ [rad/s]	*π*/2	4*π*/9	7*π*/18	*π*/3	5*π*/18	2*π*/9	*π*/6	*π*/9	*π*/18	0
*c* [Nm⋅s/rad]	0	1/*π*	2/*π*	3/*π*	4/*π*	5/*π*	6/*π*	7/*π*	8/*π*	9/*π*
*θ*_*e*_ [rad]	0	16*π*/405	32*π*/405	16*π*/135	64*π*/405	16*π*/81	32*π*/135	112*π*/405	128*π*/405	16*π*/45
*k*_e_ [N⋅m/rad]	0	6/5*π*	12/5*π*	18/5*π*	24/5*π*	6/*π*	36/5*π*	42/5*π*	48/5*π*	54/5*π*

**Fig 3:**
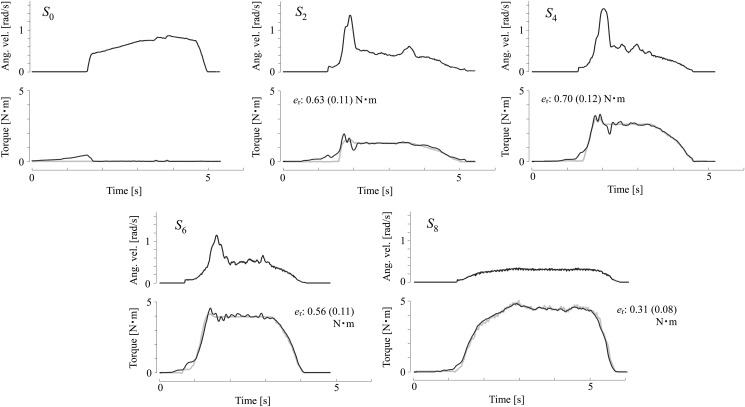
Sample resistance torques and knee angular velocities of simulated spasticity. *S*_0_: Simulated healthy knee without apparent resistance. *S*_2_ and *S*_4_: Knees with resistances for which the velocity-dependent components were obvious. *S*_6_ and *S*_8_: Knees with resistances sufficiently large to impede angular velocity. The black and gray curves respectively represent the observed and set torques, and *e*_r_ is the maximum and mean errors between the two.

Fig 3 shows examples of the behaviors of the simulated spasticity conditions. The figures show the angular velocities and resistance torques that were converted from the outputs of a force sensor that was fixed to the lower thigh link of the robot. The sample values were recorded without a wearer. As the spasticity level rose from *S*_0_ to *S*_8_, the resistance torque clearly increased. For *S*_0_, there was hardly any resistance torque apart from the static friction of the gear. For *S*_2_ and *S*_4_, the velocity-dependent resistances were noticeable, and their profiles were similar to those of the angular velocities. The values of the constant resistance *τ*_r_ for *S*_6_ and *S*_8_ were significant. The measured torques fairly matched the set torques, with the maximum error being less than 1 N⋅m.

### Results of severity rating

The ratios of the answers given by the therapists for each simulated spasticity condition are given in Table 3. No therapist indicated MAS level 4, which we intentionally excluded from the simulation as described earlier. There was a tendency to regard *S*_0_ and *S*_1_ as level 0, and approximately 30% of the answers indicated that the simulated spasticity conditions were of level 1. Contrary to our expectation, level 1 was several times assigned to *S*_0_, which was supposed to represent the case of no resistance. Based on the introspective reports obtained after the experiment, the participants considered the mass of the wearable robot to be non-negligible, and this additional mass constituted a kind of disturbance to the participants when judging the severity of the simulated symptoms. There was clear consensus on *S*_7_, *S*_8_, and *S*_9_ being level 2 conditions. In addition, the majority of the answers suggested that *S*_3_ and *S*_5_ corresponded to level 1+, whereas the answers for *S*_2_, *S*_4_, and *S*_6_ were distributed over a few levels. Overall, the results exhibited a clear trend of the MAS level increasing with increasing simulated resistance (Spearman’s rank correlation *ρ* = 0.71, and *p* = 2.2E-16).

**Table 3:** Response ratios for each simulated spasticity.

	Simulated spasticity									
MAS	*S*_0_	*S*_1_	*S*_2_	*S*_3_	*S*_4_	*S*_5_	*S*_6_	*S*_7_	*S*_8_	*S*_9_
4	0	0	0	0	0	0	0	0	0	0
3	0	0	0	6.2	0	6.2	6.2	6.2	12.5	25.0
2	6.2	6.2	6.2	18.8	37.5	12.5	31.3	62.5	81.3	75.0
1+	6.2	6.2	31.3	50.0	31.3	56.3	37.5	18.8	6.2	0
1	31.3	31.3	25.0	25.0	18.8	25.0	25.0	6.2	0	0
0	56.3	56.3	37.5	0	12.4	0	0	6.2	0	0

The criterion that was most frequently cited in the introspective reports by the participants as being most significant was the velocity-dependent resistance (seven out of the eight participants), followed by the limited range of motion (five participants), and then the static resistance or perceived heaviness over the entire movement range (four participants). Three participants noted the importance of the overall resistance that they felt. None of them mentioned the threshold angular velocity at which the velocity-dependent resistance began.

### Discussion: Consistency of answers of therapists

Here, we discuss the quality of the simulated spasticity based on the observed consistency in the answers of the participants. It should be noted that the goodness of a simulated symptom cannot be solely judged based on answer consistency because, under clinical settings, the MAS scores of assessors are not always in good agreement [22, 23]. Nonetheless, if the consistency for the simulator is as high as that expected under clinical conditions, the simulator would be validated. A significantly low consistency in the answers given by the participants regarding the simulated symptoms would indicate that the simulator is flawed.

Fleuren et al. [22] reported an interclass correlation coefficient of 0.63 for the MAS scores of three assessors regarding knee patients, whereas the value for our experiment was 0.70. Mutlu et al. [24] also reported values of 0.61–0.87 for several muscles of diplegic children, within which range our value falls.

Park et al. [11] reported that the MAS scores of their simulated elbow spasticity obtained from eight clinicians had a Fleiss’ kappa of 0.626. The values under clinical settings vary substantially between 0.16 and 0.87 [25–27] depending on the experience of the assessors, the training sessions before the experiment, and the types of patients. The Fleiss’ kappa of our results was 0.42, which is comparable to those reported in literature.

Bohannon et al. [19] also reported consistency in the MAS scores given by two physicians, represented by a Kendall’s rank correlation coefficient of 0.85. Unfortunately, this coefficient only defined the consistency in the scores given by only two assessors, and can thus not be directly compared with our results. To enable comparison, we computed the Kendall’s rank correlation coefficient for twenty-eight pairs of assessors obtained from the eight assessors that participated in our study. The mean coefficient and standard deviation were respectively 0.66 and ±0.17, which are smaller than the values reported by Bohannon et al.

As noted above, the consistency indices of the MAS ratings of the multiple assessors of our simulated spasticity are fairly within, or not significantly below, the range of those observed from previous studies, and this validates the effectiveness of our patient simulator. However, the limitations of such consistency comparisons are as described above.

## Classification of several simulated symptoms

The second type of assessment concerns the ability of a patient simulator to present the characteristic resistances of various symptoms. To investigate this ability, we conducted a classification task in which nine physical therapists were requested to categorize five different symptoms presented by our patient simulator. We then examined the goodness of the simulator in terms of the presentation of different types of symptoms.

### Five types of simulated symptoms

The five types of simulated symptoms are presented below. The symptoms are some of those that physical therapists frequently encounter clinically. The parameters for defining the symptoms were selected based on the consensus of two experienced physical therapists (coauthors of this article), such that each symptom was slightly more obvious than the one that had just been identified.

**Fig 4:**
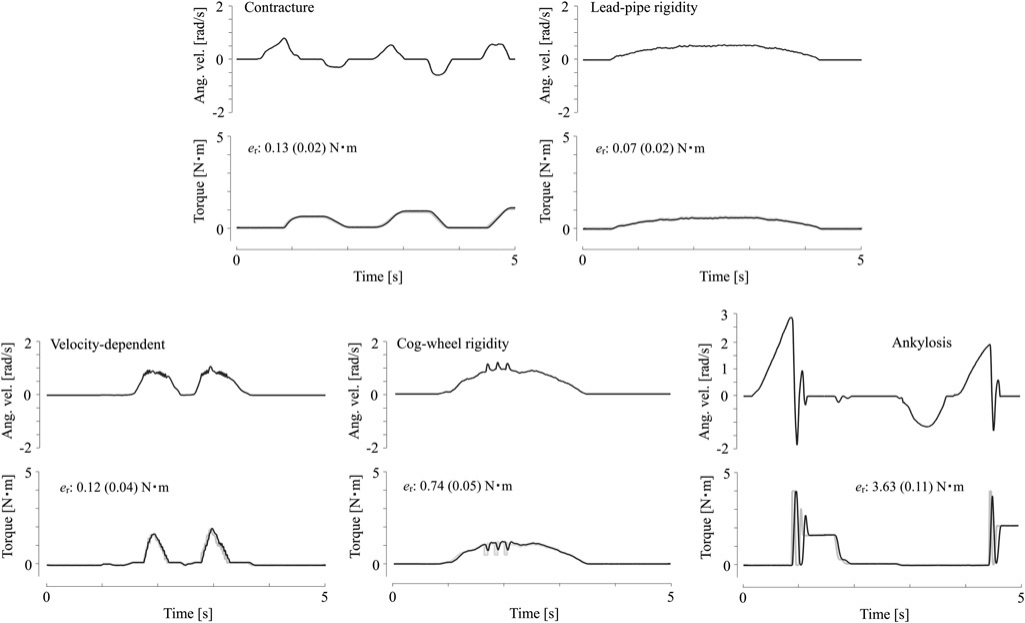
Sample resistance torques and knee angular velocities of the five types of simulated symptoms. The black and gray curves respectively represent the measured and set torques. *e*_r_ indicates the maximum and mean errors between the two values. Overall, the measured torques are in adequate agreement with the set values.

**Lead-pipe rigidity** Lead-pipe rigidity is characterized by constant stiffness of the joint during passive motion, and is often due to disorders of the basal ganglia such as Parkinson’s disease [28, 29]. By definition, we presented its resistance as a constant torque irrespective to the direction of motion. The resistance was set to 0.5 N ⋅ m. Fig 4 shows a sample of the simulated lead-pipe rigidity. As in the previous section, the data were obtained using only the wearable robot without a wearer. The dynamism of the resistance torque was minuscule, and a nearly constant torque was observed during the passive joint motion.

**Cogwheel rigidity** Cogwheel rigidity resembles lead-pipe rigidity but is distinguished by its intermittent resistance. It is also one of the typical symptoms of Parkinson’s disease [28, 29]. Because there are few available guides or literature on the simulation of this symptom, we modeled it by repetitive on-and-off resistances based on perceived realism. The torque was controlled as

$$\begin{array}{c} \tau_{\mathrm{cog}}\left(t\right)=\{\begin{array}{cc} c_{\mathrm{on}}\\ c_{\mathrm{off}} & \left(\frac{\pi }{9}<\theta <\frac{5\pi }{36},\frac{\pi }{6}<\theta <\frac{7\pi }{36},\frac{2\pi }{9}<\theta <\frac{\pi }{4}\right) \end{array} \end{array}$$ (4)

where *c*_on_ and *c*_off_ are constant torques of 1.0 and 0.5 N ⋅ m, respectively. Fig 4 shows a sample of the simulated cogwheel rigidity. Because of the limited dynamics of the DC motor, the torque of the jerk output did not precisely follow the instruction; intermittent resistances were apparent in the three jerks of the recorded torques and angular velocity.

**Spasticity (velocity-dependent resistance)** Velocity-dependent resistance or tonic stretch reflex is a feature of a spastic joint. This transient resistance can be represented by viscous resistance, as described in the previous section. Here, the resistance torque was more simply defined by

$$\begin{array}{c} \tau_{s}\left(t\right)=\{\begin{array}{cc} c_{s}\dot{\theta }\left(t\right) & \left(\dot{\theta }\left(t\right)\geq 0\right)\\ 0 & \left(\mathrm{otherwise}\right) \end{array} \end{array}$$ (5)

where *c*_s_ is the determinant of the gain relative to the angular velocity, and has a value of 1.5 Nm ⋅ s/rad. Fig 4 shows a sample simulated velocity-dependent resistance. Although the profile of the torque did not completely match that of the angular velocity, primarily because of the inertia of the lower thigh of the wearable robot, its proportionality to the angular velocity was apparent.

**Limitation of motion range (contracture)** Contracture is a limitation of joint motion range caused by a chronic shortening of soft tissues such as muscles and tendons. Abnormal elastic resistances occur at the end of the movable range. This so-called end-feel has been the focus of clinicians and is an important criterion for manual therapy and examination. We simulated the phenomenon by applying a spring resistance to the joint angle. The resistance torque was described by

$$\begin{array}{c} \tau_{\mathrm{con}}\left(t\right)=\{\begin{array}{cc} k_{\mathrm{con}}\left(\theta \left(t\right)-\theta_{\mathrm{con}}\right) & \left(\theta \left(t\right)>\theta_{\mathrm{con}}\right)\\ 0 & \left(\mathrm{otherwise}\right) \end{array} \end{array}$$ (6)

where *k*_con_ and *θ*_con_ are respectively the elastic coefficient and joint angle at which the limitation of motion manifests; *k*_con_ = 5.7 N ⋅ m/rad and *θ*_con_ = *π*/18 rad. Fig 4 shows a sample of simulated contracture. The robotic limb simply acted as a spring resistance.

**Limitation of motion range (bony ankylosis)** Another type of limitation of joint motion is caused by the deformation of joint bones or cartilages. The end-feel resistance caused by this symptom feels like a contact with nonsoft tissue or a bony contact, which is distinct from that of the previous disorder. A resistance of this nature can be simply presented by a hard virtual stick using

$$\begin{array}{c} \tau_{a}\left(t\right)=\{\begin{array}{cc} k_{a}\left(\theta \left(t\right)-\theta_{a}\right) & \left(\theta \left(t\right)>\theta_{a}\right)\\ 0 & \left(\mathrm{otherwise}\right) \end{array} \end{array}$$ (7)

where *k*_a_ is the high spring coefficient for presenting the sense of hard contact and *θ*_a_ is the joint angle at which the sticking resistance manifests; *k*_a_ = 28.5 N ⋅ m/rad and *θ*_a_ = 2*π*/3 rad. Fig 4 shows a sample limited motion range caused by bony ankylosis. The resistance torque of the simulated bony contact was impulsive and exhibited vibratory behavior after the impact. The maximum error between the set and observed torques was large, and this was clearly because of the phase difference between the torques, although their profiles were similar.

### Experiment: Tasks and participants

As in the previous experiment, a healthy person wore the wearable robot on his leg and lay on a bed in a relaxed state. The assessors were given the names of the symptoms that they would experience, and were then allowed to freely experience the five simulated symptoms for a few minutes but, without knowledge of the correspondences between the names of the symptoms and the simulated resistances. They were then presented with the five simulated symptoms sequentially, and were required to classify them in a forced-choice manner. A total of twenty-five presentations were made to each assessor in random order, with each symptom appearing five times. Nine assessors who were physical therapists, each with at least five years of clinical experience, participated in the experiment. The assessors were different from those that participated in the previous experiment and had no previous experience with similar patient simulators.

### Results of classification

The answer ratios of the experiment are given in Table 4. Each cell indicates the ratio for each combination of presented and answered symptoms.

**Table 4:** Results of the classification of the five types of simulated symptoms (mean ± standard deviation of the answer ratios of the nine assessors).

		Presented symptom				
		Lead-pipe rigidity	Spasticity	Contracture	Ankylosis	Cogwheel rigidity
	Lead-pipe rigidity	0.73 ± 0.33	0.31 ± 0.23	0.04 ± 0.09	0	0.36 ± 0.28
	Spasticity	0.11 ± 0.18	0.24 ± 0.24	0.40 ± 0.36	0	0.04 ± 0.09
Answered	Contracture	0.13 ± 0.20	0.33 ± 0.30	0.24 ± 0.19	0	0.04 ± 0.09
symptom	Ankylosis	0	0	0.07 ± 0.14	1	0
	Cog-wheel rigidity	0.02 ± 0.07	0.11 ± 0.27	0.24 ± 0.26	0	0.56 ± 0.30

The overall positive (correct) answer ratio was expected to be higher than could have been obtained by chance (0.2). The mean ratio (± standard deviation) for the nine assessors was 0.56 ± 0.097, which was higher than chance (*t*(8) = 11.0, *p* = 4.2E − 6, unpaired *t*-test). However, a consideration of the individual symptoms reveals that a few of them were not clearly classified. The answer ratios for spasticity (*t*(8) = 0.55, *p* = 0.59, unpaired *t*-test) and contracture (*t*(8) = 0.69, *p* = 0.51, unpaired *t*-test) were not significantly higher than chance, whereas those for lead-pipe rigidity (*t*(8) = 4.82, *p* = 1.3E − 3, unpaired *t*-test), cogwheel rigidity (*t*(8) = 3.60, *p* = 7.0E − 3, unpaired *t*-test), and ankylosis were above chance.

Spasticity and contracture were often confused with each other, which may be partly because spasticity or velocity-dependent resistance of actual patients is often developed together with other types of joint resistances. Although some assessors explicitly cited constant resistance and hyperexcited jerk reflective resistance (the jack-knife phenomenon) as references for lead-pipe rigidity and spasticity, respectively, the parameters that determined the perceptibility of these symptoms were set to moderate values. Classification of these symptoms was thus hindered. In addition, the sticking sensations in cogwheel rigidity were small, which impeded distinction between the two types of rigidities.

In summary, there was consensus among the experienced therapists regarding typical lead-pipe rigidity, bony ankylosis, and cogwheel rigidity. In contrast, the simulations of spasticity and contracture require improvement to enable distinction between the two. Although the parameters of the symptoms were maintained at moderate values in the experiment, increasing them would facilitate classification by assessors.

### Discussion: Limitation of classification task

Spasticity and contracture were frequently confused in the experiment. Clinicians generally should be able to distinguish symptoms to ensure that the patient receives the appropriate therapy. However, confusing symptoms are not necessarily judged solely on the basis of manual examination. Hence, classification performance cannot be regarded as the only index for evaluating a patient simulator, but it should be used in conjunction with other measures as described in the discussion section. However, it is our consideration that it would still be beneficial for a patient robot to be capable of simulating some confusing symptoms even when they do not need to be completely classified by manual examination. This is because simulators are also used to afford trainees some kind of practical experience with symptoms to complement the lessons of theoretical lectures. Such educational applications have not been thoroughly investigated, but are expected to be intensively pursued in the future.

## General Discussion

Here, we discuss a few general issues regarding the assessment of robotic patient simulators for training physical therapists. We note how the assessment methods proposed in this article should be utilized, and also discuss the use of assessments based on subjective judgment as well as other types of performance measures for patient simulators.

### Assessment measure as relative performance comparator

In industry, absolute performance indices that can be used as standards for testing products are often preferred. However, the indices used in the present study (at least the consistency of the assessor ratings) are likely to be subjected to significant variance in test institutes. As stated earlier, previous studies observed very different interclass correlations among the MAS scores of assessors. Hence, the consistency indices or average correct answer ratios of classification tasks cannot be appropriately used as absolute test standards. The assessment measures proposed in this article are more effective for comparing different patient simulators tested under the same or similar conditions, rather than for absolute judgment. They may also be used for relative testing to determine whether or not a certain product has been successfully upgraded. In this sense, the assessment measures of the present article would be the first practical performance measures for developing patient simulators.

### Subjective or objective assessment?

Measures based on physical quantities may sometimes be considered preferable to those based on subjective evaluation by therapists. Given that the purpose of a simulator is the mimicking of an actual patient, the similarity between the dynamic joint resistance patterns of simulated and actual symptoms should be used as a measure [11]. However, in physical therapy educational facilities, trainees learn characteristic symptoms using subjective criteria. From this view point, the training simulators should be evaluated based on how well they present the characteristics of impaired joints that are shared by the majority of experienced therapists.

### Other necessary assessments

Multiple criteria are usually used to assess complex systems like patient simulators. Here, we discuss required assessments other than those used in this study.

The most important feature of patient simulators is the realism of the simulated symptoms, and this was partly assessed in this study. To evaluate the realism, the qualities of the symptom models and the capabilities of the actuators also require consideration. There have been previous studies that emphasized these aspects. For example, Park et al. [11] evaluated their model in terms of its ability to mimic the joint resistances of actual patients, and Kikuchi et al. [2] used two types of actuators to compensate for the individual limitations.

The realism of other features is also important. Several previous works have advocated the implementation of the complex motions of human joint [4, 6–8]. In addition, Masutani et al. [1] highlighted the importance of the skin and bone features in their simulator.

The number of symptoms that a patient simulator can present determines its educational value, and such becomes an inevitable performance measure.

When a patient simulator is designed for training physical therapists, a rating system for the therapeutic techniques that it is used to practice is required. Hence, the goodness of the rating system would also be a factor for assessing the simulator. Further studies are required to developing such a rating system.

Finally, assistive functions for instructors should be incorporated into patient simulators used for training purposes. For example, a programmable function for tuning the simulated symptoms would be useful in educational settings.

## Conclusion

Robotic patient simulators are used to aid the training of physical therapists in manual examination techniques. Such simulators are expected to enhance the quality of training because trainees rarely have the opportunity to deal with actual patients in educational facilities. Many research groups have thus developed diverse patient simulators in recent years. However, there is no general method for evaluating the simulators. The assessment methods proposed in this article enable evaluation of the ability of simulators to present the typical and intrinsic joint resistances that characterize symptoms that are generally accepted among experienced clinicians. The more accurately the simulator presented the common features of symptoms, the higher the two types of assessment measures.

The first assessment measure was used to evaluate the ability of the simulator to present different severities of a specific disease. The assessors rated the simulated symptoms using well-known criteria, and the consistency of the ratings of the assessors was examined. The quality of the simulated symptoms was discussed based on the consistency index. If the simulator accurately presented the typical features of joint resistances, the consistency of the ratings would be high. We used the method to evaluate the spasticity simulation of a wearable patient knee simulator. Eight experienced physical therapists subjectively rated the simulated spasticity based on the MAS. The observed consistency indices were comparable to those obtained from previous clinical studies (ICC = 0.70, Fleiss’ kappa = 0.42, and Kendall’s rank correlation = 0.66).

The second assessment measure was used to evaluate the ability of the simulator to present different types of symptoms. The assessors classified the simulated symptoms based on the observed joint resistances. High correct or positive answer ratios indicated agreement among the assessors regarding the features of the simulated symptoms. We used this method to evaluate the simulations of five different symptoms by the wearable patient robot. Nine physical therapists classified the simulated symptoms into five categories. The overall positive answer ratio was 56%, which was significantly greater than chance. However, the simulated spasticity and contracture were often confused with each other, and their simulations require improvement.

The two assessment measures address different aspects of patient simulators and are to be used in a complementary manner. They are relative indices that can be used to compare different simulators, rather than absolute standards. We expect that these measures would contribute to the development of commercial patient simulators for training physical therapists in the near future.
